# Novel Haplotype Indicator for End-Stage Renal Disease Progression among Saudi Patients

**DOI:** 10.1155/2019/1095215

**Published:** 2019-08-22

**Authors:** Cyril Cyrus, Shahanas Chathoth, Chittibabu Vatte, Nafie Alrubaish, Othman Almuhanna, J. Francis Borgio, Samir Al-Mueilo, Fahd Al Muhanna, Amein K. Al Ali

**Affiliations:** ^1^Department of Biochemistry, College of Medicine, Imam Abdulrahman Bin Faisal University, Dammam, Saudi Arabia; ^2^Department of Internal Medicine, King Fahd Hospital of the University, Imam Abdulrahman Bin Faisal University, Al-Khobar, Saudi Arabia; ^3^Institute for Research and Medical Consultation, Imam Abdulrahman Bin Faisal University, Dammam, Saudi Arabia

## Abstract

**Background:**

End-stage renal disease (ESRD) is the result of hypertensive nephrosclerosis and chronic glomerular diseases and is associated with high morbidity and mortality. There are strong heritable components in the manifestation of the disease with a genetic predisposition to renal disorders, including focal segmental glomerulosclerosis and arterionephrosclerosis. Recent studies in genetics have examined modifiable risk factors that contribute to renal disease, and this has provided a deep insight into progressive kidney disease. Single-nucleotide polymorphisms at the proximity of *SHROOM3*, CST3, *SLC7A9*, and *MYH9* genes have been associated with an increased risk of developing CKD and ESRD.

**Methods:**

A total of 160 CKD patients and 189 control subjects of Saudi origin participated in the study. Eight polymorphisms (*SHROOM3-*rs9992101, rs17319721; *SLC7A9*-rs4805834; *MYH9*-rs4821480, rs4821481, rs2032487, rs3752462; CST3-rs13038305) were genotyped using TaqMan assay, and the haplotype analysis was done using the HaploView 4.2 software.

**Results:**

Haplotype analysis revealed a *novel haplotype* “*E6*”-GTTT to be associated significantly with an increased risk for ESRD (*p*=0.0001) and CKD (*p*=0.03).

**Conclusion:**

CKD is often silent until symptomatic uremia during the advanced stages of the disease. The newly identified haplotype will help recognize patients at risk for a rapid progression of CKD to ESRD. Accurate detection and mapping of the genetic variants facilitates improved risk stratification and development of improved and targeted therapeutic management for CKD.

## 1. Introduction

End-stage renal disease (ESRD) is commonly caused by hypertensive nephrosclerosis and chronic glomerular diseases [1] although a causative link between nephrosclerosis and hypertension is yet to be established [2]. Studies have examined modifiable risk factors that contribute to CKD. However, there are strong heritable components in the manifestation of the disease [3, 4], with the vast majority of individuals suffering from comorbid conditions, such as hypertension or diabetes [5, 6].

Since the advent of genome-wide association studies (GWAS), many novel loci associated with common human diseases have been identified [7], including CKD [3, 8–10]. A genetic polymorphism in shroom family member 3 (*SHROOM3*) has been identified as a CKD susceptibility locus through GWAS. *SHROOM3* is a regulator of epithelial cellular arrangement and planar remodeling [11], which contributes to glomerular filtration barrier integrity [12]. Many studies have suggested that *SHROOM3* plays an important role in mammalian kidney development and human kidney disease through estimated GFR (eGFR). One of these CKD-associated *SHROOM3* variants, rs17319721, has been shown to impact cis-expression and renal allograft fibrosis [13]. Genetic polymorphisms in solute carrier family 7-member 9 *(SLC7A9)* gene, an amino acid transporter in renal proximal tubule cells, cause cystinuria [14], showing an association with GFR [3, 15], and have been identified as a risk factor for CKD patients of European ancestry [8]. Variants of cystatin C (*CST3*) have been also shown to impact altered eGFR and kidney disease [16]. Polymorphisms in myosin heavy chain 9 (*MYH9*) gene on chromosome 22 have been shown to be associated with a risk for focal segmental glomerulosclerosis (FSGS), HIV-associated nephropathy CKD with admixed nondiabetic kidney disease, hypertension-associated ESRD, and nondiabetic etiologies of ESRD [17–20]. The *MYH9* risk polymorphisms are common among African Americans, contributing approximately 40–45% of all ESRD and 70% of nondiabetic ESRD [21].

The United States Renal Data System 2016 [22] reported the prevalence of ESRD to be 2,067 per million with an incidence rate of 370/million/year in the country. Genetic underpinnings of pediatric renal diseases, such as congenital and infantile nephrotic syndromes, are significantly higher in the Kingdom of Saudi Arabia (KSA) than in the Western world [23]. The prevalence of ESRD in KSA has exhibited a rapid increase in the past decades resulting in a rate that exceeds those seen in European and American populations [24]. The incidence and prevalence of CKD in the KSA is estimated to be approximately 1.72 million, equating to about 6% of the population. Out of these, only 7.1% are aware of their disease status, and this unawareness often results in poorer outcomes in such patients [25]. Furthermore, there is a sharp annual increase in the rate of CKD patients who develop ESRD, and this accounts for 2.21% deaths annually [24]. The prevalence of diabetic nephropathy among adult ESRD patients is 42.5% with a mortality rate of 18.6% compared to 6.9% of nondiabetic patients. While ethnicity is thought to play a large role in CKD genetics, very few genotyping studies of established CKD associations have been performed to date in Saudi Arabia and the surrounding regions. Our earlier study [26] highlighted the association of the eight SNPs in these four genetic regions and proposed a statistically significant method of predicting the CKD using FGF23, vitamin D_3_ level, and *MYH9* genotypes [26]. Haplotypes represent a combination of genetic determinants along a single chromosome that are either preserved intact or separated by recombination over time. Haplotypes are commonly used in research to identify a disease-conferring gene or locus. Presently, much interest surrounds the use of genetic association studies, which have an advisedly more powerful study design than the linkage- or family-based studies in localizing susceptibility loci for common diseases that confer moderate risk [27]. Here, we present the haplotype association of these SNPs in 160 Saudi CKD and 189 non-CKD subjects from KSA towards the CKD progression risk assessment.

## 2. Materials and Methods

### 2.1. Study Population

The study included 160 Saudi CKD patients reporting to the Department of Nephrology at the King Fahd Hospital of the University, Al Khobar. Stringent inclusion criteria for both, cases and controls, were followed due to the high prevalence rate of CKD in the Saudi population. Patients with a history of any phosphate wasting disorder, including tumor-induced osteomalacia (TIO), X-linked hypophosphatemia (XLH), autosomal dominant hypophosphatemic rickets (ADHR), and untreated primary hyperthyroidism, and those undergoing renal replacement therapy were excluded from the study. Controls consisted of 189 healthy subjects of Saudi origin without evidence of renal disorders (serum creatinine <1.4 and <1.2 mg/dl in men and women, respectively). This study was approved by the Ethical Committee of the Imam Abdulrahman Bin Faisal University and conducted according to the Declaration of Helsinki. Signed written informed consent was obtained from all participants.

### 2.2. Genotyping Assay

DNA was isolated from the blood of 349 individuals using QIAamp DNA Mini Kit (Qiagen, USA) as per the manufacturer's instructions. Genotyping analysis was performed by ABI TaqMan SNP genotyping assays on 160 CKD patients and 189 controls. Allele-specific TaqMan® PCR technology is a highly sensitive assay and was performed using ABI 7500 Fast real-time PCR. Disease-associated SNPs selected from published articles, namely, rs9992101, rs17319721 (*SHROOM3*), rs4805834 (*SLC7A9*), rs4821480, rs4821481, rs2032487, rs3752462 (*MYH9*), and rs13038305 (*CST3*), were included. An additional 100 coronary artery disease patients with hypertension were genotyped for the *MYH9* (rs4821480, rs4821481, rs2032487, and rs3752462) SNPs.

### 2.3. E Haplotyping

*MYH9* haplotypes spanning 12–23 introns were reconstructed by 4 tagging SNPs. The haplotypes comprised the four SNP loci of the *MYH9* gene in the order rs4821480 (T/g), rs4821481 (T/c), rs2032487 (C/t), and rs3752462 (C/t). The 4 SNPs were genotyped, and the haplotype blocks were determined using the HaploView 4.2 software that assigned haplotypes into chromosome-specific blocks based on the partition-ligation approach through EM algorithm [28]. To identify the nonrandom association of the eight SNPs by estimates of linkage disequilibrium (LD), each pair of SNPs was computed using the standard D-prime method. Patient population was further stratified into cohorts as CKD (eGFR > 15) and ESRD (eGFR < 15) to assess the significance of the haplotype variations.

### 2.4. Statistical Analysis

SPSS version 19 (Chicago, Illinois) was used to complete the statistical analysis. Mutation status was classified as positive or negative qualitatively. The genotype frequencies were tested for Hardy–Weinberg equilibrium among the control subjects.

## 3. Results

The present study investigated 160 CKD cases, in which 85 were males and 75 were females, with a mean age of 47.68 ± 17.27 years. The baseline characteristics of the study participants are shown in Supplementary [Supplementary-material supplementary-material-1].

TaqMan analysis for genetic variants rs9992101, rs17319721 (*SHROOM3*), rs4805834 (*SLC7A9*), rs4821480, rs4821481, rs2032487, and rs3752462 (*MYH9*), and rs13038305 (*CST3*) was carried out for all samples. The control group was consistent with Hardy–Weinberg equilibrium for all SNPs. The distribution of the analyzed genotype polymorphisms was reported in our previous study [26].

**Figure 1:**
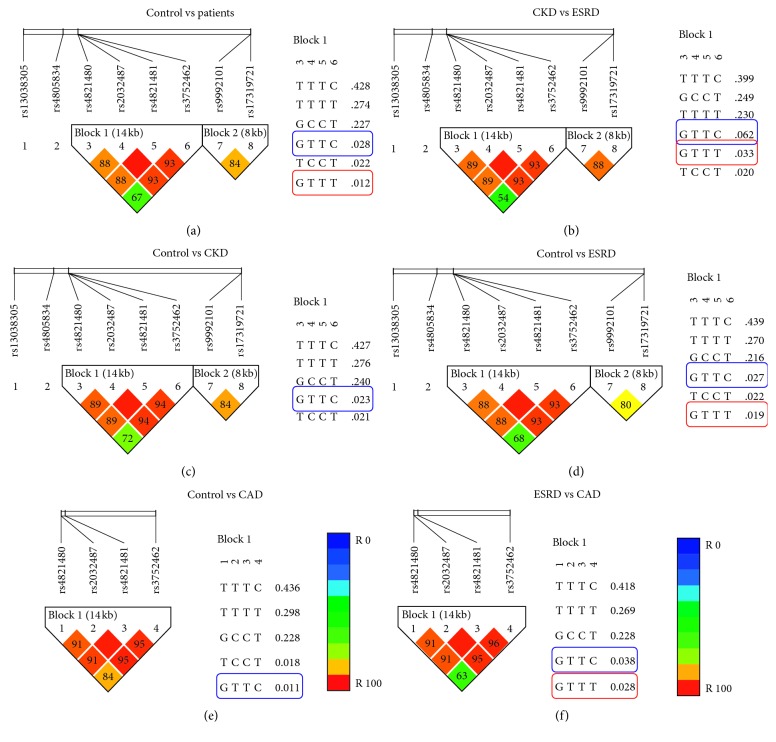
Linkage disequilibrium (LD) analysis. LD patterns between the two *SHROOM* and four *MYH9* SNPs in CKD patients vs. control cohort. The pairwise correlation between the SNPs were measured as *r*^*2*^ and are shown (×100) in each diamond. Coordinates are according to the NCBI build dbSNP 144 (r107). (a) Control vs. patients. (b) CKD vs. ESRD. (c) Control vs. CKD. (d) Control vs ESRD. (e) Control vs. CAD. (f) ESRD vs. CAD.

The HaploView analysis results are represented in Figure 1. Tables 1 and 2 indicate the frequency of various *MYH9* E and *SHROOM3* haplotypes and their association, respectively. A new haplotype “E6”-GTTT is revealed to be significantly associated with the patient cohort (*p*=0.03), stratified CKD cohort (*p*=0.002), and ESRD cohort (*p*=0.0001). The common haplotype GTTC (E4) (*p*=0.0009) was also found to be associated with CKD (Table 1). On further stratification, E6 haplotype was found to be strongly associated with ESRD (*p*=0.0001) in ESRD vs. control and in CKD vs. ESRD (*p*=0.04) analysis.

To validate the new E6 haplotype, an additional 100 CAD patients with hypertension were genotyped for the *MYH9* (rs4821480, rs4821481, rs2032487, and rs3752462) SNPs. Haplotype analysis revealed that the new haplotype was insignificant in the CAD patients. Also, the novel E6 haplotype was still found to be significantly associated (*p*=0.010) when ESRD was compared to the CAD cohort (Table 1).

## 4. Discussion

CKD is becoming an important health issue worldwide and a major cause for morbidity and mortality. Genetic searches for vital markers of CKD are essential to identify individuals who are at risk for ESRD. Numerous genes have been shown to be associated with CKD. *SHROOM3*, a regulator of epithelial cellular arrangement contributes to glomerular filtration barrier integrity while, *CST3* which is involved in creatinine and cystatin synthesis, is strongly associated with multiple kidney-related traits. The *SLC7A9* gene, which is expressed in renal proximal tubule cells, was shown to be strongly associated with the markers of kidney function, creatinine, and eGFR. *MYH9* gene encodes a protein which is expressed in the glomerular podocyte [26].

*MYH9* gene mutations result in nephritis to varying degrees in Epstein and Fechtner syndromes [21]. *MYH9* SNPs and haplotypes are associated with a risk for T2D and non-T2D nephropathy, lupus nephritis, hypertensive nephropathy, and FSGS [17, 18, 20, 29–32]. The haplotype analysis of this study shows more combinatorial importance for the *MYH9* gene region than SNPs. Oleksyk et al. [33] reported that the E1 haplotype of the *MYH9* gene region, prominent in sub-Saharan Africa, is probably involved in the increased risk of developing CKD by increasing glomerulosclerosis and proteinuria through activation of nephritis by the deregulation of podocyte function and not by immunological mechanisms. In the present study, the E1 (GCCT) haplotype lacked any association towards CKD or ESRD, contradictory to the findings of Colares et al. [34]. The rare E5 (GCTC) is the only other haplotype carrying the C allele at rs4821481, which was noted in only two of the human genome diversity project populations, with frequencies of 0.02 in Mandenka, a West African ethnic group, and 0.01 in Palestinians [33]. Kopp et al. [18] reported the E2 haplotype prominent in the European, Middle Eastern, and South and Central Asian populations to be protective against renal disease, and Tavira et al. [35] reported the same protective effect in a Spanish cohort. The E2 (TTTC) and E3 (TTTT) haplotypes lacked an association in the present study. Interestingly, the haplotype frequencies of the present study were divergent from those reported from other populations [33]. The E4 and E6 haplotypes were found to be significantly associated with an increased risk of CKD and ESRD, respectively. Even in the stratified groups, ESRD (eGFR < 15 mL/min/1.73 m^2^) and CKD (eGFR > 15 mL/min/1.73 m^2^), the E4 (GTTC) was associated with an increased risk of CKD, although the novel haplotype GTTT (*p* = 0.0001) was found to be only associated with ESRD.

The SNPs rs9992101 and rs17319721 located in the *SHROOM3* gene on chromosome 4q21 are closely associated with CKD [8], and the former is in high linkage disequilibrium (LD) with rs17319721. The rs17319721 (A) allele is associated with increased *SHROOM3* transcription and is therefore associated with an increased glomerular filtration rate, thereby increasing the risk for CKD [3]. Both these SNPs and their haplotypes were not associated with a risk of CKD in the present study.

## 5. Conclusion

CKD is often silent until the advanced stages of the disorder. Thus, many individuals remain unaware until symptomatic uremia is detected, and they begin to suffer from renal ailments. The newly identified haplotype will help identify the patients at risk for quicker progression of CKD towards ESRD. This study will further our understanding of the biological mechanisms of kidney function by identifying loci which may potentially influence metabolic renal functions.

## Figures and Tables

**Table 1:** Frequency of *MYH9* E haplotypes compared between stratified groups such as CKD, ESRD, and CAD.

Groups	Haplotype	Frequency	Chi square	*p* value
Control vs. patients	TTTC	0.43	1.075	0.2998
	TTTT	0.27	1.461	0.2268
	GCCT	0.23	0.339	0.5603
	GTTC	0.03	10.979	**9 × 10** ^**−4**^
	TCCT	0.02	0	0.9923
	GTTT	0.01	4.663	**0.0308**
	TTT	0.7	5.295	**0.0214**
	GCC	0.24	0.391	0.532
	GTT	0.04	15.934	**6.6 × 10** ^**−5**^
	TCC	0.02	0	0.9896

Control vs. CKD	TTTC	0.43	1.718	0.19
	TTTT	0.28	1.625	0.2024
	GCCT	0.24	2.613	0.106
	GTTC	0.02	9.492	**0.0021**
	TCCT	0.02	0.07	0.7919
	TTT	0.7	7.108	**0.0077**
	GCC	0.25	2.584	0.1079
	GTT	0.03	10.934	**9 × 10** ^**−4**^
	TCC	0.02	0.067	0.7954

CAD vs. ESRD	TTTC	0.418	0.009	0.9256
	TTTT	0.269	3.632	0.0567
	GCCT	0.228	0.551	0.4581
	GTTC	0.038	8.051	**0.0045**
	GTTT	0.028	6.615	**0.0101**
	TCCT	0.015	0.46	0.4975

Control vs. ESRD	TTTC	0.44	0.294	0.5876
	TTTT	0.27	2.968	0.0849
	GCCT	0.22	0.084	0.7723
	GTTC	0.03	15.431	**8.6 × 10** ^**−5**^
	TCCT	0.02	0.022	0.8828
	GTTT	0.02	14.495	**1 × 10** ^**−4**^
	TTT	0.71	5.181	**0.0228**
	GCC	0.22	0.06	0.8066
	GTT	0.05	30.498	**3.34 × 10** ^**−5**^
	TCC	0.02	0.021	0.8838

CKD vs. ESRD	TTTC	0.4	0.517	0.4723
	GCCT	0.25	2.542	0.1108
	TTTT	0.23	0.26	0.6104
	GTTC	0.06	0.494	0.4821
	GTTT	0.03	4.217	**0.04**
	TCCT	0.02	0.008	0.9302
	TTT	0.63	0.082	0.7747
	GCC	0.26	2.442	0.1181
	GTT	0.09	3.329	0.0681
	TCC	0.02	0.007	0.9338

CAD vs. Control	TTTC	0.436	0.533	0.4656
	TTTT	0.298	0.214	0.644
	GCCT	0.228	0.369	0.5435
	TCCT	0.018	0.933	0.3342
	GTTC	0.011	0.154	0.6946

**Table 2:** Frequency of *SHROOM3* haplotypes compared between patient stratified by CKD, ESRD, and control cohorts.

Groups	Haplotype	Frequency	Chi square	*p* value
Control vs. patients	GG	0.69	0.507	0.4765
	AA	0.24	1.525	0.2168
	GA	0.04	0.035	0.8512
	AG	0.03	1.91	0.1669

Control vs. CKD	GG	0.69	0.563	0.4531
	AA	0.24	2.098	0.1475
	GA	0.04	0.002	0.963
	AG	0.03	2.383	0.1227

Control vs. ESRD	GG	0.69	0.824	0.3639
	AA	0.23	0.155	0.6939
	GA	0.05	1.998	0.1575
	AG	0.04	0.052	0.8203

CKD vs. ESRD	GG	0.66	0.014	0.9052
	AA	0.27	0.807	0.369
	GA	0.05	1.49	0.2222
	AG	0.02	1.514	0.2186

## Data Availability

The datasets used and/or analyzed during the current study are available from the corresponding author.

## Supplementary Materials

Supplementary Materials Supplementary Table 1: baseline characteristics of the total study population and stagewise distribution.
