# Supplementary material for: Novel Haplotype Indicator for End-Stage Renal Disease Progression among Saudi Patients
Source: Int J Nephrol. 2019 Aug 22;2019:1095215. doi: 10.1155/2019/1095215 (PMC6724424; doi:10.1155/2019/1095215)
Supplement: Supplementary Materials — Supplementary Table 1: baseline characteristics of the total study population and stagewise distribution. [file 1095215.f1.docx]

**Supplementary Table 1:** Baseline characteristics of the total study population and stage wise distribution.

|  | | Total CKD Cases | STAGE 3 | STAGE 4 | STAGE 5 |
| --- | --- | --- | --- | --- | --- |
| Number of subjects | | 160 | 64 | 20 | 76 |
| Age, years (Mean±SD) | | 47.68± 17.27 | 41.92 ± 15.47 | 52.15 ± 16.48 | 51.34 ± 17.75 |
| Gender, Male (%) | | 85 (53%) | 27 (42%) | 10 (50%) | 48 (63%) |
| eEGFR (ml/min/1.73m2) | | 17.4 ± 25.1 | 40.2 ± 3.5 | 21.8 ± 4.8 | 7.5 ± 3.280 |
| PTH (pmol/L) | | 37.05 ± 59.35 | 11.44 ± 4.69 | 22.03 ± 10.59 | 62.58 ± 78.36 |
| Serum phosphorus (pg/mL) | | 4.14 ± 1.09 | 3.65 ± 0.63 | 4.18 ± 0.71 | 4.54 ± 1.29 |
| Serum Calcium (mg/dl) | | 9.08 ± 5.95 | 8.65 ± 0.51 | 12.26 ± 16.67 | 8.62 ± 1.15 |
| Albumin (g/dl) | | 3.38 ± 0.56 | 3.52 ± 0.54 | 3.47 ± 0.56 | 3.23 ± 0.55 |
| Vitamin D3 (ng/mL) | | 22.32 ± 12.65 | 22.22 ± 14.21 | 19.34 ± 9.05 | 23.18 ± 12.06 |
| Creatinine (mg/dl) | | 5.27 ± 4.76 | 0.97 ± 0.47 | 2.86 ± 0.95 | 9.52 ± 3.45 |
| ALP (u/L) | | 158.89 ± 245.36 | 92.05 ± 64.91 | 135.90 ± 133.68 | 221.24 ± 334.17 |
| FGF23 (pg/ml) | | 485.64 ± 802.58 | 62.01 ± 46.76 | 210.87 ± 432.83 | 914.69 ± 977.98 |
| Cause of Renal Failure | LN | 45 | 40 | 2 | 3 |
|  | HTN | 37 | 8 | 4 | 25 |
|  | GN | 17 | 4 | 2 | 11 |
|  | DM | 57 | 10 | 12 | 35 |

LN: Lupus nephritis; HTN: Hypertension; GN: Glomerular nephritis; DM: Diabetes. All biochemical parameters are represented as Mean±SD; Cause of renal failure was not known for 4 cases
